# Unidentified Inert Ingredients in Pesticides: Implications for Human and Environmental Health

**DOI:** 10.1289/ehp.9374

**Published:** 2006-08-18

**Authors:** Caroline Cox, Michael Surgan

**Affiliations:** 1 Northwest Coalition for Alternatives to Pesticides, Eugene, Oregon, USA; 2 Center for Environmental Health, Oakland, California, USA; 3 Office of the Attorney General of New York State, Environmental Protection Bureau, New York, New York, USA

**Keywords:** ecologic effects, exposure, formulations, inert ingredients, pesticides, toxicology

## Abstract

**Background:**

By statute or regulation in the United States and elsewhere, pesticide ingredients are divided into two categories: active and inert (sometimes referred to as other ingredients, adjuvants, or coformulants). Despite their name, inert ingredients may be biologically or chemically active and are labeled inert only because of their function in the formulated product. Most of the tests required to register a pesticide are performed with the active ingredient alone, not the full pesticide formulation. Inert ingredients are generally not identified on product labels and are often claimed to be confidential business information.

**Objectives:**

In this commentary, we describe the shortcomings of the current procedures for assessing the hazards of pesticide formulations and demonstrate that inert ingredients can increase the toxicity of and potential exposure to pesticide formulations.

**Discussion:**

Inert ingredients can increase the ability of pesticide formulations to affect significant toxicologic end points, including developmental neurotoxicity, genotoxicity, and disruption of hormone function. They can also increase exposure by increasing dermal absorption, decreasing the efficacy of protective clothing, and increasing environmental mobility and persistence. Inert ingredients can increase the phytotoxicity of pesticide formulations as well as the toxicity to fish, amphibians, and microorganisms.

**Conclusions:**

Pesticide registration should require full assessment of formulations. Evaluations of pesticides under the National Environmental Policy Act, the Endangered Species Act, and similar statutes should include impact assessment of formulations. Environmental monitoring for pesticides should include inert ingredients. To enable independent research and risk assessment, inert ingredients should be identified on product labels.

Pesticides are toxic chemicals that are both ubiquitous and unique. Unlike other toxic chemicals, they are designed to kill, repel, or otherwise harm living organisms [U.S. Environmental Protection Agency (EPA) 2005c], and they are one of the few toxic substances that are intentionally applied to the environment [National Research Council (NRC) 1993]. Monitoring programs conducted in the United States have found pesticides in “one or more samples from every stream sampled” (Gilliom et al. 2006), in > 70% of common foods [U.S. Department of Agriculture (USDA) 2006], and in over half of adults and children (Centers for Disease Control and Prevention 2005).

In the United States, the regulatory system for pesticides differs from other toxic chemical regulatory programs. Under the Federal Insecticide, Fungicide, and Rodenticide Act (FIFRA 2002), active ingredients—those which “prevent, destroy, repel, or mitigate any pest”—are subject to greater scrutiny than inert (or sometimes other) ingredients (U.S. EPA 1997). The combination of active and inert ingredients, as marketed and used, is called a formulation (U.S. EPA 2006b). Most countries surveyed by the Organization of Economic Co-operation and Development (OECD) make a similar distinction, although terminology can be different: “adjuvants” and “formulants” are sometimes used to describe inert ingredients, and formulations can be called “preparations” (OECD 1994, 1998).

In ordinary usage, the word “inert” refers to something that is physically, chemically, or biologically inactive. The U.S. EPA recognizes that the statutory nomenclature for pesticides under FIFRA engenders public misunderstanding, stating that “many consumers have a misleading impression of the term ‘inert ingredient,’ believing it to mean water or other harmless ingredients” (U.S. EPA 1997). In fact, an inert ingredient “may have biological activity of its own, it may be toxic to humans, and it may be chemically active” (U.S. EPA 2002). The arbitrary distinction between active and inert ingredients is well illustrated by the > 500 inert ingredients that, according to the U.S. EPA (2006a), have been or are currently used as active ingredients.

A significant proportion of typical pesticide formulations are inert ingredients. In a survey of over 200 common household products in retail stores in Oregon, the Northwest Coalition for Alternatives to Pesticides (NCAP 2006b) found that these products contained on average 86% inert ingredients. Similar results were found in surveys of products for sale in New York in 1990, 1997, and 1999 (Surgan and Gershon 2000). Agricultural products also contain a significant proportion of inert ingredients. In a review of over 100 agricultural products, NCAP (2006a) found that they contained an average of > 50% inert ingredients.

Inert ingredients serve a variety of functions in pesticide formulations, acting as solvents, surfactants, or preservatives, among many other functions (U.S. EPA 2002, 2005a). Products with the same active ingredient may be described as granular, flowable, emulsifiable, or wettable based on the inert ingredients in the formulation [National Pesticide Telecommunications Network (NPTN) 1999]. A single product may contain a number of inert ingredients, each with a different purpose in the formulation (U.S. EPA 2005b).

Independent assessment of the hazards of pesticide formulations is stymied by the lack of public access to product-specific information about inert ingredients. FIFRA (2002) requires that active ingredients be identified on product labels, but makes no such requirement for inert ingredients. The only products for which complete identification of inert ingredients is required are minimum risk, FIFRA-exempt products (U.S. EPA 2005a, Part 158.25). As a result, inert ingredients are rarely identified on the product label. In 1999, only 10% of > 100 commonly available pesticide products sold in retail stores in New York identified any of the inert ingredients on the label. None of these labels identified all of the inert ingredients in the products (Surgan and Gershon 2000).

Pesticide manufacturers claim that some inert ingredient information is protected as confidential business information (NCAP v. Browner 1996). In addition, inert ingredients are protected as confidential by many governments (OECD 1998). Our experience is that the current process for identifying ingredients involves substantial bureaucratic delay and, in some instances, litigation. There is a clear need for more public disclosure of the identity of all ingredients of pesticide products.

## Inadequate Assessment of the Hazards of Pesticide Formulations

The U.S. EPA (2004) has identified almost 3,000 substances, with widely varying toxicity, that are used as inert ingredients in the United States. For example, paper is used as an inert ingredient, but so are toxic chemicals such as naphthalene and xylene (U.S. EPA 2004). Also, about 50% of all inert ingredients are at least moderately risky (U.S. EPA 2002). Given the toxicity of inert ingredients and their widespread use in pesticide products, formulations should be fully assessed when pesticides are registered with the U.S. EPA. This, however, is not currently the case. Of the 20 toxicologic tests required (or conditionally required) to register a pesticide in the United States, only 7 short-term acute toxicity tests use the pesticide formulation; the rest are done with only the active ingredient. The medium-and long-term toxicity tests that explore end points of significant concern (cancer, reproductive problems, and genetic damage, for example) are conducted with the active ingredient alone. The requirements for other types of tests are similar. Only half of the required (or conditionally required) tests of environmental fate use the formulated product, as do only a quarter of the tests for effects on wildlife and nontarget plants (U.S. EPA 2005a, Parts 158.290, 158.340, 158.490, and 158.540). As a result, many potential long-term effects of pesticide formulations are not assessed as part of the registration process. Testing requirements are similar in many other countries (OECD 1994).

Research indicates that some inert ingredients in pesticide formulations can significantly affect the human health and environmental impacts of these products. Published studies detailed below demonstrate that tests conducted with active ingredients alone are inadequate as the basis for the evaluation of the health and environmental impacts of pesticide formulations.

## Inert Ingredients Can Increase Toxicity of Pesticide Formulations

Numerous studies indicate that inert ingredients may enhance the toxicity of pesticide formulations to the nervous system, the cardiovascular system, mitochondria, genetic material, and hormone systems.

A household formulation of the insecticide bifenthrin reduced the viability of rodent nerve cell cultures, whereas bifenthrin did not. Both the formulation and the active ingredient reduced the outgrowth of neuritis *in vitro*, but the effects of the formulation were more severe (Tran et al. 2006). These observations suggest that the inert ingredients would enhance developmental neurotoxic effects of bifenthrin.

Inert ingredients can also be toxic to the cardiovascular system. An herbicidal formulation of glufosinate caused a decrease in blood pressure and changes in heart rate of rats, *in vivo* (Koyama et al. 1997). Glufosinate alone had no effects on either parameter. Similar results were obtained *in vitro.*

Oakes and Pollack (1999) reported that inert ingredients of three herbicides also increased *in vitro* inhibition of mitochondrial oxidative activity. The concentration of active ingredient required to reduce mitochondrial activity by 50% was 136 times higher for a formulation containing only 2,4-D (2,4-dichloro-phenoxyacetic acid) and picloram than the concentration of those ingredients required when the inert ingredients were also included. These authors found similar results with a formulation containing 2,4-D and 2,4,5-T (2,4,5-trichlorophenoxyacetic acid) (Oakes and Pollack 2000). Peixoto (2005) found that a glyphosate formulation caused a significant reduction in the activity of rat liver mitochondrial respiratory complexes *in vitro* but that glyphosate alone had no effect.

Pesticide formulations have proven to be more potent genotoxins than active ingredients alone in a variety of test systems. *In vitro* treatment of human lymphocytes with glyphosate and a glyphosate formulation resulted in a significantly higher rate of induction of sister chromatid exchange by the formulated product (Bolognesi et al. 1997). Both the formulation and glyphosate increased micronucleus formation in mouse bone marrow; the increase was “more pronounced” with the formulation. Zeljezic et al. (2006) found that an herbicidal formulation containing atrazine increased DNA damage in human lymphocytes but atrazine alone did not.

Inert ingredients may enhance the reproductive toxicity of active ingredients. Both the herbicide glyphosate and a glyphosate formulation were toxic to human placenta cell cultures (Richard et al. 2005). However, the formulation was significantly more toxic than glyphosate alone; the median lethal dose for the formulation was half that of the active ingredient.

Several reports demonstrate disruption of endocrine function by inert ingredients. In one study, a glyphosate-containing herbicide formulation inhibited progesterone production *in vitro* in mouse Leydig cells, but glyphosate did not (Walsh et al. 2000). Richard et al. (2005) noted that a glyphosate formulation inhibited the activity of human placental cell aromatase, which converts androgens into estrogens. Again, glyphosate alone did not inhibit the activity of this enzyme. In another study, Lin and Garry (2000) found that two 2,4-D formulations caused estrogen-like proliferation of MCF-7 breast cancer cells *in vitro*, whereas 2,4-D did not.

## Inert Ingredients Can Increase Exposure to Pesticide Formulations

Inert and active ingredients can interact to diminish the protective efficacy of both clothing and skin, reduce the efficacy of washing, and increase persistence and off-target movement of pesticides.

Dermal exposure is the most common exposure route for people who handle or apply pesticides. Some inert ingredients can increase dermal absorption or penetration of the active ingredient. In a comparison of the penetration of three formulated herbicidal products through hairless mouse skin with their respective active ingredients, Brand and Mueller (2002) found that dermal penetration of the formulations was 3–30 times greater than the penetration of the active ingredients alone. Similar results were obtained in studies of absorption of the insecticide lindane and the wood preservative pentachlorophenol through human and porcine skin, respectively (Baynes et al. 2002; Dick et al. 1997a, 1997b). In all three of these studies, solvents used as inert ingredients increased the dermal absorption of the active ingredient. A surfactant used as an inert ingredient increased absorption of the insecticide carbaryl through porcine skin (Baynes and Riviere 1998).

Pesticide labels often instruct users to wear protective gloves or clothing to reduce the potential for exposure to toxic ingredients. The efficacy of protective clothing, however, may be diminished by inert ingredients in the formulation; as a result, pesticide workers may be unable to make a fully informed decision. For example, solvents used as “inerts” in a formulation of the herbicide 2,4-D act as cosolvents to increase the permeation of the active ingredients through nitrile gloves (Harville and Que Hee 1989). Similar cosolvent effects occurred when a formulation of the herbicide 4-chloro-2-methylphenoxyacetic acid was tested on four glove materials (Purdham et al. 2001).

Inert ingredients can also reduce the protective efficacy of work clothing that is washed and reused, thereby enhancing exposure to pesticides. Some inert ingredients adversely affected laundry removal of the insecticide methyl parathion from clothing. Laughlin et al. (1985) found that the emulsifiable concentrate formulation was more difficult to remove than a wettable powder and an encapsulated formulation. Similar results were obtained in a comparison of emulsifiable concentrate and wettable powder formulations of the insecticides cyfluthrin and cypermethrin (Laughlin et al. 1991).

It is not surprising that some inert ingredients can increase persistence of pesticides in the environment; that could be the reason for their inclusion in the formulation. However, increasing persistence also results in more potential for human and other nontarget exposure. Montemurro et al. (2002) reported that the persistence of the insecticide chlorpyrifos in soil, foliage, and fruit varied significantly among formulations containing different inert ingredients. A microencapsulated formulation was most persistent. Inert ingredients can also affect the distribution and behavior of active ingredients in the environment, in some instances enhancing runoff, leaching, and volatilization. Wilson et al. (1995) found that herbicide runoff from nursery containers varied between granular and sprayable formulations. The concentration of the insecticide imidacloprid in runoff from turf treated with a granular formulation was twice as high as the concentration following treatment with a wettable powder formulation (Ambrust and Peeler 2002).

Inert ingredients can even affect volatilization of active ingredients, contributing to airborne migration and inhalation exposures. Volatilization of the insecticide azadirachtin varied among formulations; volatilization was greater from a wettable powder formulation than from three emulsifiable concentration formulations (Sundaram 1997).

## Inert Ingredients Can Increase Ecotoxicity of Pesticide Formulations

The severity of varied toxic effects of active ingredients of pesticides in nontarget plants, animals, and microorganisms can be enhanced by the inert ingredients with which they are formulated.

Adverse impacts of pesticides on nontarget plants can be mediated by inert ingredients in the formulation. For example, in a phytotoxic compound formed by thermal degradation of a fungicidal benomyl formulation containing starch as an inert ingredient, the degradation is facilitated by the starch, a source of water for the reaction (Tang and Song 1996). Kohmann (1999) found that an inert ingredient in a permethrin-based insecticide product reduced frost tolerance of spruce (*Picea abies*) seedlings. Inert ingredients may also compound interactions between active ingredients. The herbicides glyphosate and glufosinate-ammonium were shown to be synergistically phytotoxic with the herbicide metsulfuron-methyl, and this synergy was more pronounced for formulations than for active ingredients alone (Kudsk and Mathiassen 2004).

Inert ingredients can increase avian toxicity of some pesticide formulations. Treatment of chick embryos with a 2,4-D formulation resulted in a significantly higher frequency of sister chromatid exchanges than did treatment with 2,4-D alone (Arias 2003).

Toxic effects of some pesticide formulations on fish can be increased by the inert ingredients. One of the most commonly known examples is glyphosate; some formulations are 10–100 times more acutely toxic to fish than is the active ingredient alone (U.S. EPA 1993). Kiparissis (2003) found that a formulation of the fungicide vinclozolin, but not vinclozolin alone, caused fish (*Oryzias latipes*) to develop intersex gonads. In another study, Arsenault et al. (2004) reported that exposure of captive salmon (*Salmo salar* L.) to environmentally relevant levels of the inert surfactant 4-nonylphenol reduced the growth of smolts, suggesting that exposure to a formulation containing 4-nonylphenol might explain the decline of some wild salmon populations.

Similarly, amphibians may be adversely affected by inert ingredients. In a study by Swann et al. (1996), two formulations of the insecticide chlorpyrifos were more neurotoxic *in vitro* to frogs and caused more damage (swelling) to mitochondria than chlorpyrifos alone. Howe et al. (2004) found that exposure of *Rana pipiens* tadpoles to environmentally relevant concentrations of glyphosate formulations reduced size at metamorphosis but increased time to metamorphosis, frequency of tail damage, and frequency of abnormal gonads. Glyphosate alone did not have these effects.

Pesticide formulations can be strikingly more toxic to microorganisms than their active ingredients alone. Everett and Dickerson (2003) found that a glyphosate formulation was 100 times more toxic to ciliated protozoans than glyphosate. Garcia-Ortega et al. (2006) reported that a formulation of the insecticide propetamphos was 100 times more toxic to the microbial flora in sediments than propetamphos alone.

## Discussion

There is a substantial and growing body of research that demonstrates the inadequacy of reliance on testing the active ingredient alone when assessing the exposure to pesticides, their toxic effects, and their behavior in the environment. Inert ingredients are often biologically or chemically active and can affect each of these parameters. Demonstrations of important impacts of inert ingredients have not been limited to particular classes of pesticides, types of formulations, or toxicity end points. Instead, it appears that the effects of inert ingredients may be both common and far-reaching.

It is often unclear if inert ingredients are directly responsible for certain toxic effects or if those effects are attributable to interactions between inert and active ingredients. Because inert ingredients are rarely identified, studies comparing the effects of the active ingredient, the inert ingredients, and the formulation are not common. Such three-way comparisons were performed in six cited studies (Koyama et al. 1997; Oakes and Pollack 1999, 2000; Swann et al. 1996; Tang and Song 1996; Zeljezic et al. 2006); this literature suggests that the situation is complex. In three instances, interactions between active and inert ingredients were important (Oakes and Pollack 2000; Swann et al. 1996; Tang and Song 1996); three studies demonstrated that the increased toxicity was primarily due to the inert ingredients (Koyama et al. 1997; Oakes and Pollack 1999; Zeljezic et al. 2006).

Similarly, full assessment of exposure to pesticide formulations is impeded by the lack of information about the concentration of individual inert ingredients. Only five of the cited studies provided this information (Dick et al. 1997b; Howe 2004; Koyama et al. 1997; Oakes and Pollack 2000; Zeljezic et al. 2006). Label disclosure of all ingredients with percent composition would facilitate these much-needed studies.

Consistent with our growing awareness of the complexities of the toxicity of mixtures, it is no surprise that pesticide formulations act differently than active ingredients alone. As early as 1988, the National Research Council (NRC 1988) concluded: “Mixtures that are of particular concern include chemicals generated in fire, hazardous wastes, pesticides, drinking water, fuels and fuel combustion products,” adding that “toxicological studies of mixtures are essential for estimating human risks.”

More recently, the Agency for Toxic Substances and Disease Registry identified chemical mixtures as one of six priority areas in public health research (de Rosa et al. 2004). In Europe and Japan, Feron et al. (2002) found “a growing interest among toxicologists and regulators in the toxicology and risk assessment of chemical mixtures.” A review of ecotoxicology tools identified mixtures as one of the top three challenges in assessing environmental contamination (Eggen and Segner 2003).

Current testing requirements for pesticides are inadequate to fully assess the health and environmental effects of these mixtures. To remedy this situation, all pesticide ingredients should be identified on product labels, and pesticide registration should be based on full assessments of formulations as they are sold and used. Requirements that manufacturers develop analytical methods for active ingredients should be expanded to include inert ingredients. Furthermore, evaluations of pesticides required under the National Environmental Policy Act (1969), the Endangered Species Act (1973), analogous state laws, and similar laws in other countries should consider all impacts of formulations, not just those of active ingredients. Programs to track pesticide use should include both active and inert ingredients, as should monitoring of pesticides in humans and the environment. Researchers could then use this information to set priorities.

In 1994, the American Medical Association (1997) urged the U.S. Congress, government agencies, and other organizations to “support all efforts to list both active and inert ingredients on pesticide container labels and material safety data sheets.” Health and environmental researchers worldwide should support such efforts. Independent investigation is stymied by the secrecy that shrouds the inert ingredients in pesticide products.

## References

[b1-ehp0114-001803] Ambrust KL, Peeler HB (2002). Effects of formulation on the runoff of imidacloprid from turf. Pest Manag Sci.

[b2-ehp0114-001803] American Medical Association (1997). Educational and informational strategies to reduce pesticide risks. Prev Med.

[b3-ehp0114-001803] Arias E (2003). Sister chromatid exchange induction by the herbicide 2,4–dichlorophenoxyacetic acid in chick embryos. Ecotoxicol Environ Saf.

[b4-ehp0114-001803] Arsenault JTM, Fairchild WL, MacLatchy DL, Burridge L, Haya K, Brown SB (2004). Effects of water-borne 4-nonylphenol and 17B-estradiol exposures during parr-smolt transformations on growth and plasma IGF-I of Atlantic salmon (*Salmo salar* L.). Aquat Toxicol.

[b5-ehp0114-001803] Baynes RE, Brooks JD, Mumtaz M, Riviere JE (2002). Effect of chemical interactions in pentachlorophenol mixtures on skin and membrane transport. Toxicol Sci.

[b6-ehp0114-001803] Baynes RE, Riviere JE (1998). Influence of inert ingredients in pesticide formulations on dermal absorption of carbaryl. Am J Vet Res.

[b7-ehp0114-001803] Bolognesi C, Bonatti S, Degan P, Gallerani E, Peluso M, Rabboni R (1997). Genotoxic activity of glyphosate and its technical formulation Roundup. J Agric Food Chem.

[b8-ehp0114-001803] Brand RM, Mueller C (2002). Transdermal penetration of atrazine, alachlor, and trifluralin; effect of formulation. Toxicol Sci.

[b9-ehp0114-001803] Centers for Disease Control and Prevention 2005. Third National Report on Human Exposure to Environmental Chemicals. Available: http://www.cdc.gov/exposurereport/3rd/default.htm [accessed 30 April 2006].

[b10-ehp0114-001803] de Rosa CT, El-Masri HA, Pohl H, Cibulas W, Mumtaz MM (2004). Implications of chemical mixtures in public health practice. J Toxicol Environ Health B Crit Rev.

[b11-ehp0114-001803] Dick IP, Blain PG, Williams FM (1997a). The percutaneous absorption and skin distribution of lindane in man. I. *In vivo* studies. Hum Exp Toxicol.

[b12-ehp0114-001803] Dick IP, Blain PG, Williams FM (1997b). The percutaneous absorption and skin distribution of lindane in man. II. *In vitro* studies. Hum Exp Toxicol.

[b13-ehp0114-001803] Eggen RIL, Segger H (2003). The potential of mechanism-based bioanalytical tools in ecotoxicological exposure and effect assessment. Anal Bioanal Chem.

[b14-ehp0114-001803] Endangered Species Act 1973. 16USC1531. Available: http://www.access.gpo.gov/uscode/title16/chapter35_.html [accessed 28 September 2006].

[b15-ehp0114-001803] Everett KDE, Dickerson HW (2003). *Ichthyophthirius multifilis* and *Tetrahymena thermophila* tolerate glyphosate but not a commercial herbicidal formulation. Bull Environ Contam Toxicol.

[b16-ehp0114-001803] Feron VJ, Cassee FR, Groten JP, van Vliet PW, van Zorge JA (2002). International issues on human health effects of exposure to chemical mixtures. Environ Health Perspect.

[b17-ehp0114-001803] FIFRA (Federal Insecticide, Fungicide and Rodenticide Act) 2002. 7USC136. Available: http://www.access.gpo.gov/uscode/title7/chapter6_subchapterii_.html [accessed 28 September 2006].

[b18-ehp0114-001803] Garcia-Ortega S, Holliman PJ, Jones DL (2006). Toxicology and fate of Pestenal and commercial propetamphos formulations in river and estuarine sediment. Sci Total Environ.

[b19-ehp0114-001803] Gilliom RJ Barbash JE Crawford CG Hamilton PA Martin JD Nakagaki N et al. 2006. The Quality of Our Nation’s Waters: Pesticides in the Nation’s Streams and Ground Water, 1992–2001 Reston, VA:U.S. Geological Survey. Available: http://pubs.usgs.gov/circ/2005/1291/ [accessed 28 September 2006].

[b20-ehp0114-001803] Harville J, Que Hee SS (1989). Permeation of a 2,4-D isooctyl ester formulation through neoprene, nitrile, and tyvek protection materials. Am Ind Assoc Hyg J.

[b21-ehp0114-001803] Howe CM (2004). Toxicity of glyphosate-based pesticides to four North American frog species. Environ Toxicol Chem.

[b22-ehp0114-001803] Kiparissis Y, Metcalfe TL, Balch GC, Metcalfe CD (2003). Effects of the antiandrogens, vinclozolin and cyproterone acetate on gonadal development in the Japanese medaka (*Oryzias latipes*). Aquat Toxicol.

[b23-ehp0114-001803] Kohmann K (1999). Side-effects of formulations of permethrin and fenvalerate insecticides on frost resistance and filed performance of *Picea abies* seedlings. Scand J Forest Res.

[b24-ehp0114-001803] Koyama K, Koyama K, Goto K (1997). Cardiovascular effects of a herbicide containing glufosinate and a surfactant: *in vitro* and *in vivo* analyses in rats. Toxicol Appl Pharmacol.

[b25-ehp0114-001803] Kudsk P, Mathiassen SK (2004). Joint action of amino acid biosynthesis-inhibiting herbicides. Weed Res.

[b26-ehp0114-001803] Laughlin J Easley C Gold RE 1985. Methyl parathion residue in contaminated fabrics after laundering. In: Dermal Exposure Related to Pesticide Use: Discussion of Risk (Honeycutt RC, Zweig G, Ragsdale NN, eds). Washington DC:American Chemical Society, 177–187.

[b27-ehp0114-001803] Laughlin J, Newburn K, Gold RE (1991). Pyrethroid insecticides and formulations as factors in residues remaining in apparel fabrics after laundering. Bull Environ Contam Toxicol.

[b28-ehp0114-001803] Lin N, Garry VF (2000). In vitro studies of cellular and molecular developmental toxicity of adjuvants, herbicides, and fungicides commonly used in Red River Valley, Minnesota. J Toxicol Environ Health A.

[b29-ehp0114-001803] Montemurro N, Grieco F, Lacertosa G, Visconti A (2002). Chlorpyrifos decline curves and residue levels from different commercial formulations applied to oranges. J Agric Food Chem.

[b30-ehp0114-001803] National Environmental Policy Act 1969. 42USC4321. Available: http://www.access.gpo.gov/uscode/title42/chapter55_.html [accessed 28 September 2006].

[b31-ehp0114-001803] NCAP (Northwest Coalition for Alternatives to Pesticides) 2006a. Inert Ingredients in Common Agricultural Pesticide Products. Available: http://www.pesticide.org/agriculturalinerts.html [accessed 18 April 2006].

[b32-ehp0114-001803] NCAP (Northwest Coalition for Alternatives to Pesticides) 2006b. Inert Ingredients in Common Household Pesticide Products. Available: http://www.pesticide.org/householdinerts.html [accessed 18 April 2006].

[b33-ehp0114-001803] NCAP (Northwest Coalition for Alternatives to Pesticides) v. Browner 1996. Case No. 94–1100, U.S. District Court for the District of Columbia, Washington, DC.

[b34-ehp0114-001803] NPTN (National Pesticide Telecommunications Network) 1999. Pesticide Formulations. Available: http://npic.orst.edu/factsheets/formulations.pdf [accessed 1 May 2006].

[b35-ehp0114-001803] NRC (National Research Council) 1988. Complex Mixtures –Methods for in Vivo Toxicity Testing. Washington, DC:National Academy Press. 25032282

[b36-ehp0114-001803] NRC (National Research Council) 1993. Soil and Water Quality: An Agenda for Agriculture. Washington, DC:National Academy Press.

[b37-ehp0114-001803] Oakes DJ, Pollak JK (1999). Effects of a herbicide formulation, Tordon 75D, and its individual components on the oxidative functions of mitochondria. Toxicology.

[b38-ehp0114-001803] Oakes DJ, Pollak JK (2000). The in vitro evaluation of the toxicities of three related herbicide formulations containing ester derivatives of 2,4,5-T and 2,4-D using sub-mitochondrial particles. Toxicology.

[b39-ehp0114-001803] OECD (Organization for Economic Co-operation and Development) 1994. Data Requirements for Pesticide Registration in OECD Member Countries: Survey Results. Available: http://www.olis.oecd.org/olis/1994doc.nsf/LinkTo/ocde-gd(94)47 [accessed 3 August 2006].

[b40-ehp0114-001803] OECD (Organization for Economic Co-operation and Development) 1998. OECD Governments’ Approaches to the Protection of Proprietary Rights and Confidential Business Information in Pesticide Registration. Available: http://www.olis.oecd.org/olis/1998doc.nsf/LinkTo/env-mc-chem(98)20 [accessed 2 August 2006].

[b41-ehp0114-001803] Peixoto F (2005). Comparative effects of the Roundup and glyphosate on mitochondrial oxidative phosphorylation. Chemosphere.

[b42-ehp0114-001803] Purdham JT, Menard BJ, Bozek PR, Sass-Kortsak AM (2001). MCPA permeation through protective gloves. Appl Occup Environ Hyg.

[b43-ehp0114-001803] Richard S, Moslemi S, Sipahutar H, Benachour N, Serllini G-E (2005). Differential effects of glyphosate and Roundup on human placental cells and aromatase. Environ Health Perspect.

[b44-ehp0114-001803] Sundaram KMS (1997). Effect of additives in the neem formulations on deposition, volatilization and persistence of azadirachtin in spruce foliage. J Environ Sci Health B.

[b45-ehp0114-001803] Surgan MH Gershon AG 2000. The Secret Ingredients in Pesticides: Reducing the Risk. New York:Office of the Attorney General, Environmental Protection Bureau. Available: http://www.oag.state.ny.us/press/reports/inerts/table_of_contents.html [accessed 2 April 2006].

[b46-ehp0114-001803] Swann JM, Schultz TW, Kennedy JR (1996). The effects of the organophosphorus insecticides Dursban and Lorsban on the ciliated epithelium of the frog palate *in vitro*. Arch Environ Contam Toxicol.

[b47-ehp0114-001803] Tang CS, Song L-W (1996). Spontaneous *N,N*’-dibutylurea (DBU) formation in Benlate DF formulation under elevated temperatures. Arch Environ Contam Toxicol.

[b48-ehp0114-001803] Tran V, Hoffman N, Mofunanaya A, Pryor SC, Ojugbele O, McLaughlin A (2006). Bifenthrin inhibits neurite outgrowth in differentiating PC12 cells. Med Sci Monitor.

[b49-ehp0114-001803] USDA 2006. Pesticide Data program: Annual Summary Calendar Year 2004. Washington DC:U.S. Department of Agriculture. Available: http://www.ams.usda.gov/science/pdp/Summary2004.pdf [accessed 2 April 2006].

[b50-ehp0114-001803] U.S. EPA 1993. Registration Eligibility Decision (RED): Glyphosate. Washington, DC:U.S. Environmental Protection Agency. Available: http://www.epa.gov/oppsrrd1/REDs/old_reds/glyphosate.pdf [accessed 25 April 2006].

[b51-ehp0114-001803] U.S. EPA (U.S. Environmental Protection Agency) 1997. Pesticide Regulation Notice 97–6. Use of Term “Inert” in the Label Ingredients Statement. Available: http://www.epa.gov/opppmsd1/PR_Notices/pr97-6.html [accessed 14 April 2006].

[b52-ehp0114-001803] U.S. EPA (U.S. Environmental Protection Agency) 2002. The Office of Pesticide Program’s Guidance Document on Methodology for Determining the Data Needed and the Types of Assessments necessary to make FFDCA Section 408 Safety Determinations for Lower Toxicity Pesticide Chemicals. Available: http://www.epa.gov/oppfead1/cb/csb_page/updates/lowertox.pdf [accessed 12 April 2006].

[b53-ehp0114-001803] U.S. EPA (U.S. Environmental Protection Agency) 2004. Inert (other) Pesticide Ingredients in Pesticide Products -Categorized List of Inert (Other) Pesticide Ingredients. Available: http://www.epa.gov/opprd001/inerts/lists.html [accessed 12 April 2006].

[b54-ehp0114-001803] U.S. EPA (U.S. Environmental Protection Agency) 2005a. Data Requirements for Registration. 40CFR158. Available: http://www.access.gpo.gov/nara/cfr/waisidx_05/40cfr158_05.html [accessed 28 September 2006].

[b55-ehp0114-001803] U.S. EPA (U.S. Environmental Protection Agency) 2005b. Inert (other) Ingredients in Pesticide Products. Available: http://www.epa.gov/opprd001/inerts/ [accessed18 April 2006].

[b56-ehp0114-001803] U.S. EPA (U.S. Environmental Protection Agency) 2005c. What Is a Pesticide? Available: http://www.epa.gov/pesticides/about/index.htm [accessed 12 April 2006].

[b57-ehp0114-001803] U.S. EPA (U.S. Environmental Protection Agency) 2006a. Substance Registry System. Available: http://www.epa.gov/srs/ [accessed 12 April 2006].

[b58-ehp0114-001803] U.S. EPA (U.S. Environmental Protection Agency) 2006b. Terms of Environment: Glossary, Abbreviations and Acronyms. Glossary: F. Available: http://www.epa.gov/OCEPAterms/fterms.html [accessed 11 April 2006].

[b59-ehp0114-001803] Walsh LP, McCormick C, Martin C, Stocco DM (2000). Roundup inhibits steroidogenesis by disrupting steroidogenic acute regulatory (StAR) protein expression. Environ Health Perspect.

[b60-ehp0114-001803] Wilson PC, Whitwell T, Riley MB (1995). Effects of ground cover and formulation on herbicides in runoff water from miniature nursery sites. Weed Sci.

[b61-ehp0114-001803] Zeljezic D, Garaj-Vrhovac V, Perkovic P (2006). Evaluation of DNA damage induced by atrazine and atrazine-based herbicide in human lymphocytes in vitro using a comet and DNA diffusion assay. Toxicol In Vitro.

